# Hand dermatitis: a 6-year experience in a tertiary referral Brazilian hospital^☆^

**DOI:** 10.1016/j.abd.2024.08.010

**Published:** 2025-03-29

**Authors:** Larissa Relva da Fonte Gonçalves Endlich, Luciana Paula Samorano, Ricardo Spina Nunes, Vitor Manoel Silva dos Reis

**Affiliations:** Department of Dermatology, Hospital das Clínicas, Faculty of Medicine, Universidade de São Paulo, São Paulo, SP, Brazil

**Keywords:** Dermatitis, allergic contact, Dermatitis, irritant, Dermatitis, occupational, Eczema, Eczema, dyshidrotic, Hand dermatoses

## Abstract

**Background:**

Hand dermatitis (HD) is a prevalent inflammatory skin disease with a significant socioeconomic impact.

**Objectives:**

To characterize the population of HD patients followed up at the Department of Dermatology of a tertiary hospital.

**Methods:**

A cross-sectional, retrospective and descriptive study was carried out through the analysis of medical records of HD patients assisted at the Allergy Clinic of the Department of Dermatology, Hospital das Clínicas, Faculdade de Medicina, Universidade de São Paulo, between March 1, 2016 and August 31, 2022.

**Results:**

Of the 175 patients, 73.3% were women, and the mean age at the onset of the condition was 41.7 years. There was a statistically significant association between occupation categories and the presence of occupational dermatitis. Cases were classified as irritant contact dermatitis (49.7%), allergic contact dermatitis (57.1%) and endogenous vesicular HD (3.4%). It was observed a statistically significant higher frequency of patch tests positivity for methylchloroisothiazolinone and methylisothiazolinone (MCI/MI) and nickel sulfate in women and for potassium bichromate and carba mix in men. DLQI was assessed in 77 patients, and the average score was 7.8 points.

**Study limitations:**

As limitations, the authors point out data collection from medical records, which lacked some information. Furthermore, as this was a cross-sectional study, it was not possible to assess cause-and-effect relationships between the variables.

**Conclusions:**

The present data reinforces the importance of patch tests in HD investigation and highlights the high sensitivity rates to MCI/MI and nickel sulfate in Brazilian women and to potassium bichromate and carba mix in Brazilian men patients.

## Introduction

Hand dermatitis is an inflammatory skin disease that usually begins in the third decade of life.^1^ It presents a lifetime prevalence of 14.5% in the general population, and a pooled incidence rate of 7.3 cases/1000 person-years.^1^ The condition is responsible for more than 80% of all occupational dermatitis.^2^, ^3^

Subtypes of exogenous hand eczema include irritant contact dermatitis (ICD) and allergic contact dermatitis (ACD).^3^ The former consists of a nonspecific skin reaction to contact with a toxic or irritating chemical product, which may occur as early as the first exposure.^2^, ^3^, ^4^ On the other hand, ACD is a delayed type IV immune reaction to contact with an allergen in a sensitized individual.^5^ Its diagnosis is confirmed when there is a positive patch test reaction to the substance and a relevant exposure, documented or suspected, to the same.^3^, ^4^, ^5^

Endogenous vesicular hand dermatitis, formerly known as pompholyx or dyshidrotic eczema, is a form of endogenous disease that presents a poorly understood etiology.^3^ It manifests with recurrent vesicular eruptions on the hands, usually symmetrical and pruritic, in the absence of relevant contact allergies or irritants that could cause hand dermatitis.^2^, ^3^, ^5^

As hand eczema tends to be multifactorial, it is generally not possible to identify and eliminate the causative factor in order to cure the condition.^4^, ^6^ In addition to behavioral measures, treatment involves topical and systemic options, depending on the severity of the disease.^7^ It should be started as early as possible, once resistance to topical treatment is commonly seen in chronic disease.^4^, ^6^

Overall, the prognosis for the condition is poor.^3^ In a 15-year follow-up of hand eczema, 44% of the patients reported symptoms during the previous year, and 12% reported continuous symptoms during the entire follow-up period.^8^ Regarding occupational disease, another investigation has found that only 19.3% of patients reported complete eczema remission after five years.^9^

Hand dermatitis is a highly prevalent disease with a negative physical, psychological, social, and economic impact.^6^, ^10^, ^11^ It generates direct and indirect treatment costs due to working hours lost.^3^, ^12^

The present study aimed to characterize the population of hand dermatitis assisted at the Department of Dermatology of a tertiary hospital in São Paulo, Brazil. The authors have collected epidemiological and clinical data from hand dermatitis patients and described their disease subtypes and patch test results.

## Methods

### Study design and subjects

A cross-sectional, retrospective, and descriptive study was carried out through the analysis of the medical records of hand dermatitis patients, followed up at the Allergy Clinic of the Department of Dermatology, Hospital das Clínicas, Faculdade de Medicina, Universidade de São Paulo (HC-FMUSP). The local ethics committee approved the investigation (approval protocol number: CAAE 58151222.0.0000.0068).

Patients with hand dermatitis followed up between March 1^st^, 2016 and August 31^th^, 2022 were included in the analyses. The diagnosis of hand dermatitis was clinically established, excluding differential diagnoses, and supported by complementary exams if applicable. Exclusion criteria included patients who did not present hand dermatitis, patients with eczematous lesions on other locations than hands and feet, and those who did not perform the contact test or whose test showed inconclusive results, beyond the patients with incomplete medical records.

### Medical records evaluations

Patients' medical records were evaluated to obtain the following data: age at the onset of hand dermatitis; age at the first visit at the outpatient clinic; sex; occupation; smoking habits; diagnosis of atopic dermatitis (AD) according to Hanifin and Rajka criteria^13^; other comorbidities; subtype of hand dermatitis and its characteristics at the dermatological examination; results of patch tests; and treatments prescribed during follow-up.

In addition, Dermatology Quality of Life Index (DLQI) scores were collected from the medical records, according to the questionnaire applied within 30 days of the patient's first visit to the outpatient clinic.^14^ Hongbo and colleagues (2005) have proposed the following banding system for the DLQI score: 0‒1 Indicates no effect on patient’s Quality of Life (QOL); 2‒5 Means a small effect; 6‒10 A moderate effect; 11‒20 A very large effect; and 21‒30 Indicates an extremely large effect on patient's QOL.^15^

Occupational hand eczema is an inflammatory skin disease of the hands caused or aggravated by occupational exposure.^12^ In this study, occupational allergic contact dermatitis was identified when the patient presented a positive patch test result for occupational exposure.^12^ On the other hand, an occupational irritant contact dermatitis was defined when there were no positive patch test results for occupational exposure but there was relevant occupational exposure for irritants.^12^

### Patch tests

Patch tests have followed the Brazilian Group for Studies on Contact Dermatitis’ recommendations.^16^ The 30 substances of Brazilian Standard Battery (supplied by Endo-Derme Formulas Magistrais Ltda, São Paulo, Brazil) were standardized and positioned in the containers and applied, preferably, in the upper dorsal region of the patient.^15^ According to the exposure history, the Cosmetics Battery (supplied by Endo-Derme Formulas Magistrais Ltda, São Paulo, Brazil) was also applied.^16^

The tests were removed 48 hours after application for the first reading (D2). The second reading was performed 96 hours after the tests were applied (D4). Results were interpreted according to the clinical manifestation at the test site: negative (-); doubtful (?); mild reaction (+); strong reaction (++); and very strong reaction (+++).^17^ The test was considered suggestive of sensitization by the substance when there was a result (++) or (+++) in D4.^17^ However, in the case of positivity’s intensity reduction between D2 and D4, the test was considered negative, suggestive of primary irritation.^16^ A positive result was considered relevant when there was documented or suspected exposure to the substance.^3^, ^4^, ^5^

### Statistical analysis

Qualitative and quantitative variables were analyzed through absolute and relative frequencies and by the mean, median, standard deviation, minimum and maximum values, 25^th^ and 75^th^ percentiles (quantitative variables). Data normality was assessed by the Kolmogorov-Smirnov test. Comparison between two independent groups was performed by Student's *t*-test (normal distribution data) and the Mann-Whitney test (data without normal distribution), while the comparison between three groups was evaluated by the Kruskal-Wallis test. Association between qualitative variables was performed by Pearson's Chi-Square test or Fisher's exact test. Data were analyzed using SPSS software for Windows v.25, adopting a 5% significance level.

## Results

During the investigation time, 188 hand dermatitis patients were assisted at the Allergy Clinic of the Department of Dermatology of HC-FMUSP. After applying the exclusion criteria, 175 patients were included in the study.

Among the patients included, 129 (73.7%) were female. The mean age at onset of clinical manifestations was 41.7 years (range 2.9‒74.2) and the mean age at the beginning of outpatient follow-up was 45.5 years (range 13.8‒75.5). The mean time between the onset of symptoms and the beginning of outpatient follow-up was 46.0 months (range 0‒360).

The declared occupations were grouped into categories. The most frequent occupational category was the one related to domestic care (Table 1). In the sample, 107 patients (61.1%) were classified as presenting an occupational disease.

**Table 1:** Occupations of study’s participants grouped into categories, Department of Dermatology of HC-FMUSP, March 2016 to August 2022.

Occupational category	Declared occupations	n = 174 (n_missing_ = 1); n (%)	Occupational dermatitis	
			No (n = 67); n (%)	Yes (n = 107); n (%)
Occupations related to domestic care	Homemakers, elderly caregivers and domestic workers.	43 (24.7)	6 (14.0)	37 (86.0)
Beauty professionals	Hairdressers, barbers, beauticians, manicurists and massage therapists.	13 (7.5)	1 (7.7)	12 (92.3)
Healthcare professionals	Doctors, nurses, nursing assistants, orthopedic immobilization and orthoses technicians, community health agents, physiotherapists, dentists and surgical instrumentators.	17 (9.8)	5 (29.4)	12 (70.6)
Industry and mechanical metallurgy professionals	Chemical industry and metallurgist employees.	5 (2.9)	1 (20.0)	4 (80.0)
Construction-related occupations	Bricklayers, painters and plumbers.	16 (9.2)	4 (25.0)	12 (75.0)
Professionals with cleaning and tidying functions in non-domestic environments	Janitors and cleaners.	17 (9.8)	1 (5.9)	16 (94.1)
Professionals involved with food preparation and service in non-domestic environments	Cooks and butlers.	8 (4.6)	4 (50.0)	4 (50.0)
Professionals with administrative functions and/or who work in companies, commerce, telecommunications or transport services	Human resources professionals, sales supervisors, cashiers, logistics assistants, attendants, auditors, receptionists, sellers, administrative assistants, merchants, drivers, telecommunications installers, stockists, graphic designers and flyer distributors.	32 (18.4)	27 (84.4)	5 (15.6)
Rural professionals	Rural workers.	1 (0.6)	0	1 (100)
Artists	Craftsmen, printmakers, photographers, circus performers and dressmakers.	6 (3.4)	2 (33.3)	4 (66.7)
Occupations related to education	Teachers and students.	9 (5.2)	9 (100)	0
Retired and inactive professionals	Retirees, unemployed professionals and those temporarily away from work activities.	7 (4.0)	7 (100)	0

HC-FMUSP, Hospital das Clínicas, Faculdade de Medicina, Universidade de São Paulo.

There was a statistically significant association between occupation categories and hand dermatitis as occupational dermatitis (p < 0.001). As demonstrated in Table 1, the highest prevalence of occupational disease was observed in professionals with cleaning and tidying functions in non-domestic environments (n = 16, 94.1%), beauty professionals (n = 12, 92.3%), and occupations related to domestic care (n = 37, 86.0%).

Concerning smoking habits, 72% (n = 103; n total = 143) had never smoked, 13.3% had stopped the habit before starting follow-up at the outpatient clinic and 14.7% were active smokers.

In the sample, 12 patients (6.9%) had AD, 18 (10.3%) presented a diagnosis of psychiatric disorders (depression, anxiety and schizophrenia) and 21 (12.0%) had asthma or allergic rhinitis.

According to clinical manifestations, patients were divided into two groups: dyshidrosiform dermatitis (predominant presentation with vesicles) and non-dyshidrosiform dermatitis. In the first group, 74 patients (42.3%) were included, and in the other one, 101 participants (57.7%). Lesions on the palms and back of the hands were present in 75.4% (n = 132) and 43.1% (n = 75) of the patients, respectively, with 26.9% of the patients in the study (n = 47) presenting lesions in both locations. In addition, 36.0% (n = 63) of the patients manifested lesions on the feet.

Thirty-three hand dermatitis patients (18.9%) underwent anatomopathological examination, which results supported the diagnosis of eczema. One hundred and twenty-six patients (72.0%) had a positive patch test, of which 100 were considered relevant. Thus, 57.1% of the patients in the study had a positive and relevant patch test, while 14.9% presented a positive irrelevant result and 28.0% had a negative patch test.

Based on patch test results and clinical manifestations, patients were classified into ICD (n = 69, 39.4%), ACD (n = 82, 46.8%), ICD and ACD simultaneously (n = 18, 10.3%), and endogenous vesicular HD (n = 6, 3.4%). The antigens most frequently associated with relevant positive patch tests were nickel sulfate, methylchloroisothiazolinone, and methylisothiazolinone (MCI/MI ‒ Kathon CG) and fragrance mix (25.7%, 24.0% and 14.3%, respectively) (Table 2).

**Table 2:** Patch test results for patients with hand dermatitis followed up at the Department of Dermatology of HC-FMUSP, March 2016 to August 2022.

Tested substances		Relevant positive patch tests (n = 175); n (%)	Relevant positive patch tests by sex		p-value
			Male (n = 46); n (%)	Female (n = 129; n (%)	
Peru balsam	Yes	6 (3.4)	0	6 (4.7)	0.342^a^
	No	169 (96.6)	46 (100)	123 (95,3)	
PPD mix	Yes	5 (2.8)	0	5 (3.9)	0.328^a^
	No	170 (97.1)	46 (100)	124 (96.1)	
Hydroquinone	Yes	2 (1.1)	1 (2.2)	1 (0.8)	0.458^a^
	No	173 (98.8)	45 (97.8)	128 (99.2)	
Potassium bichromate	Yes	12 (6.8)	7 (15.2)	5 (3.9)	**0.015**^a^
	No	163 (93.1)	39 (84.8)	124 (96.1)	
Propylene glycol	Yes	1 (0.6)	1 (2.2)	0	0.263^a^
	No	174 (99.4)	45 (97.8)	129 (100)	
Neomycin	Yes	4 (2.3)	2 (4.3)	2 (1.6)	0.283^a^
	No	171 (97.7)	44 (95.7)	127 (98.4)	
MCI/MI	Yes	42 (24.0)	6 (13.0)	36 (27.9)	**0.043**^b^
	No	133 (76.0)	40 (87.0)	93 (72.1)	
Cobalt chloride	Yes	11 (6.3)	4 (8.7)	7 (5.4)	0.482^a^
	No	164 (93.7)	42 (91.3)	122 (94.6)	
Thiuram mix	Yes	8 (4.6)	3 (6.5)	5 (3.9)	0.435^a^
	No	167 (95.4)	43 (93.5)	124 (96.1)	
Ethylenediamine	Yes	1 (0.6)	0	1 (0.8)	0.999^a^
	No	174 (99.4)	46 (100)	128 (99.2)	
Fragrance mix	Yes	25 (14.3)	4 (8.7)	21 (16.3)	0.207^b^
	No	150 (85.7)	42 (91.3)	108 (83.7)	
MBT mix	Yes	1 (0.6)	1 (2.2)	0	0.263^a^
	No	174 (99.4)	45 (97.8)	129 (100)	
Quinoline mix	Yes	1 (0.6)	0	1 (0.8)	0.999^a^
	No	174 (99.4)	46 (100)	128 (99.2)	
Epoxy resin	Yes	1 (0.6)	1 (2.2)	0	0.263^a^
	No	174 (99.4)	45 (97.8)	129 (100)	
Thimerosal	Yes	17 (9.7)	5 (10.9)	12 (9.3)	0.775^b^
	No	158 (90.3)	41 (89.1)	117 (90.7)	
Turpentine	Yes	5 (2.9)	1 (2.2)	4 (3.1)	0.999^a^
	No	170 (97.1)	45 (97.8)	125 (96.9)	
Carba mix	Yes	8 (4.6)	5 (10.9)	3 (2.3)	**0.030**^a^
	No	167 (95.4)	41 (89.1)	126 (97.7)	
Promethazine	Yes	3 (1.7)	1 (2.2)	2 (1.6)	0.999^a^
	No	172 (98.3)	45 (97.8)	127 (98.4)	
Nickel sulfate	Yes	45 (25.7)	6 (13.0)	39 (30.2)	**0.022**^b^
	No	130 (74.3)	40 (87.0)	90 (69.8)	
Colophonium	Yes	8 (4.6)	3 (6.5)	5 (3.9)	0.435^a^
	No	167 (95.4)	43 (93.5)	124 (96.1)	
Paraphenylenediamine	Yes	7 (4.0)	0	7 (5.4)	0.192^a^
	No	168 (96.0)	46 (100)	122 (94.6)	
Formaldehyde	Yes	7 (4.0)	1 (2.2)	6 (4.7)	0.677^a^
	No	168 (96.0)	45 (97.8)	123 (95.3)	
Germall 115	Yes	1 (0.6)	1 (2.2)	0	0.263^a^
	No	174 (99.4)	45 (97.8)	129 (100)	
Tosylamide-formaldehyde resin	Yes	8 (4.6)	0	8 (6.2)	0.113^a^
	No	167 (95.4)	46 (100)	121 (93.8)	
Triethanolamine	Yes	12 (6.8)	2 (4.3)	10 (7.8)	0.734^a^
	No	163 (93.1)	44 (95.7)	119 (92.2)	
Bronopol	Yes	3 (1.7)	0	3 (2.3)	0.567^a^
	No	172 (98.3)	46 (100)	126 (97.7)	
Chlorhexidine	Yes	1 (0.6)	0	1 (0.8)	0.999^a^
	No	174 (99.4)	46 (100)	128 (99.2)	
Coconut fatty acid diethanolamide	Yes	2 (1.1)	1 (2.2)	1 (0.8)	0.458^a^
	No	173 (98.8)	45 (97.8)	128 (99.2)	

HC-FMUSP, Hospital das Clínicas, Faculdade de Medicina, Universidade de São Paulo.

p-value demonstrates the association between the variables sex and positivity in the patch test for each substance evaluated.

a Fisher's exact test.

b Pearson's Chi-Square test.

Women had a higher positivity for MCI/MI (n = 36, 27.9%) and nickel sulfate (n = 39, 30.2%) compared to men (n = 6, 13.0%, for both antigens), and this association was statistically significant (Table 2). On the other hand, male patients presented higher positivity for potassium bichromate (n = 7, 15.2%) and carba mix (n = 5, 10.9%), compared to females (n = 5, 3.9 %, and n = 3, 2.3%, respectively), also with statistical significance (Table 2).

Patients with dyshidrosiform eczema had positive patch tests for MCI/MI in 37.8% of cases (n = 28), in comparison with positivity of 13.9% (n = 14) in patients without dishydrosiform eczema (p < 0.001). In addition, a higher percentage of patch test positivity for nickel sulfate and paraphenylenediamine was observed among patients with dyshidrosiform eczema compared with non-dyshidrosiform eczema: 35.1% (n = 26) vs. 18.9% (n = 19) and 8.1% (n = 6) vs. 1.0% (n = 1), with p = 0.015 and p = 0.043, respectively.

DLQI was assessed in 77 patients, and the average score was 7.8 points. No statistically significant differences were observed between DLQI scores and the patient’s epidemiological, clinical, and treatment characteristics.

Regarding disease treatment, emollients were prescribed to all patients and topical corticosteroids to 174 (99.4%) of them. Topical tacrolimus was used in four patients (2.3%), while urea cream was administered in five cases (2.9%). Antibiotics were prescribed orally for 13 cases (7.4%) and topically for 17 patients (9.7%). Oral antihistamines were used in 140 cases (80%). Fifty-four patients (30.9%) have received cycles of oral prednisone to optimize clinical control. Methotrexate was administered to three patients to control recalcitrant disease. In the series, 43 (24.6%) patients were discharged from the outpatient clinic for regular control of clinical manifestations.

Among 87 patients with ICD, 62 were women (71.3%). The most frequent occupational category was domestic care (n = 21, 24.1%), followed by administrative functions (n = 18, 20.7%). Most of these cases (n = 68, 78.2%) presented clinically as non-dyshidrosiform eczema, compared to 37.5% (n = 33) of patients with other diagnoses – ACD and endogenous vesicular hand dermatitis (p < 0.001). Lesions were mostly located on the palms (n = 60, 69.0%), followed by the back of the hands (n = 34, 39.1%) and involvement of the feet were observed in 28 patients (32.2%). The frequency of lesions on the palms was lower in the group of ICD than in the group of other diagnoses (n = 72, 81.8%) and this association was statistically significant (p = 0.048). Twenty patients (23.0%) with ICD used oral prednisone, compared to 34 patients (38.6%) of the ones with other diagnosis (p = 0.025). Furthermore, among the patients who were discharged due to regular control of the condition, 65.1% (n = 28) had ICD, compared with 44.7% (n = 59), of those who were not discharged (p = 0.020).

One hundred patients were classified as ACD, 80% of which were women. Among ACD patients, the most frequent occupational category was domestic care (n = 27, 27.0%), followed by administrative functions (n = 19, 19.0%), and beauty professionals (n = 11, 11.0%). Most of the cases (n = 53, 53.0%) presented clinically as dyshidrosiform eczema, compared to only 28.0% (n = 21) of patients with other diagnoses (p = 0.001). Regarding the topography of the lesions, 81.0% (n = 81) of the patients manifested lesions on the palms (n = 81), 49.0% (n = 49) on the back of the hands and 37.0% (n = 37) also presented involvement of the feet. The frequency of injuries on the back of the hands was higher in this group (n = 49, 49.5%) compared to the ones with other diagnosis (n = 26, 34.7%) with statistical significance (p = 0.050). Oral prednisone was prescribed to 40.0% of these individuals during follow-up, compared to only 18.7% of patients with other diagnoses (p = 0.002). Among the patients who were discharged due to regular control of the condition, only 32.6% (n = 14) had ACD, compared with 65.2% (n = 86), of those who were not discharged (p < 0.001).

Six patients had endogenous vesicular hand dermatitis. Four of these patients (66.7%) were discharged due to regular control of the condition.

## Discussion

The present study reveals the six-and-a-half-year experience with hand dermatitis at a dermatology-specialized clinic at a tertiary Brazilian hospital. In the sample, 73.3% of the patients were female, with a mean age of 42 years at the onset of clinical manifestations. Female sex has been considered a risk factor for hand eczema development, which is probably due to a higher exposure to triggers, as predominantly female occupations, related to aesthetics, cleanliness, and health, involve frequent hand contact with humidity and hygiene products.^1^, ^18^, ^19^, ^20^ It should also be considered that women probably most often seek medical care.^21^ The gender distribution and the mean age at hand eczema onset were similar in the present study and in a recently published Brazilian investigation.^20^

Concerning the relatively long mean time (46-months) between the beginning of the condition and the beginning of outpatient follow-up, it possibly reflects patients’ delay in seeking medical care for not considering hand dermatitis a disease or even because they are not aware of the treatment options.^3^, ^22^ It is also possible that the condition is not valued or accurately diagnosed by some healthy professionals.^3^, ^22^

It was a statistically significant association between occupation categories and occupational dermatitis diagnosis, with the highest prevalence of occupational disease in professionals with cleaning and tidying functions in non-domestic environments (94.1%), beauty professionals (92.3%), occupations related to domestic care (86.0%), Industry and mechanical metallurgy professionals (80.0%), civil construction professionals (75.0%) and healthcare professionals (70.6%). These data are in agreement with the recent Brazilian literature.^20^, ^21^ An increase in hand dermatitis incidence is usually associated with wet work, possibly due to impairment of the skin barrier.^3^, ^18^, ^21^, ^23^ In addition, some occupations are frequently exposed to allergic and irritating triggers. A systematic review with meta-analysis demonstrated a significantly increased risk of contact dermatitis in healthcare professionals, hairdressers, factory workers, painters, metallurgists, and cleaners.^23^

The average DLQI score was 7.8 points, which means a moderate effect on patients’ QOL, similar to previous studies' results.^15^, ^22^, ^24^, ^25^, ^26^ It should be considered, however, that this tool may not be sufficient to analyze the magnitude of QOL impairment in hand dermatitis patients, since seriously impacted aspects, such as work capacity, are poorly represented, while other aspects less affected by the disease are valued.^17^

Only 6.9% patients in this sample had AD, which may have been underestimated due to the absence of the disease’s clinical manifestations during the appointments and the lack of patients’ accuracy to identify a previous condition. The exclusion of patients with eczematous lesions on other locations than hands and feet may also explain the lower frequency of AD patients. AD is considered an important risk factor for the development of hand dermatitis.^1^, ^3^, ^18^, ^19^, ^20^, ^27^, ^28^ In the cohort study conducted by Koskelo et al. (2022), AD was confirmed in 72.4% of hand eczema patients, while in the meta-analysis by Quaade et al. (2021) the pooled proportion of adults with current or previous hand dermatitis and a history of AD was 34.4%.^1^, ^19^

The authors have observed that women had a higher positivity frequency for MCI/MI and nickel sulfate in patch tests, while male patients presented higher positivity for potassium bichromate and carba mix. The main sensitizers found in the present study are in agreement with the literature.^20^, ^21^, ^29^, ^30^, ^31^, ^32^, ^33^, ^34^, ^35^, ^36^

MCI/MI is used as a preservative in cosmetics, detergents, water-based paints, and industrial products. Therefore, sensitization occurs mainly in domestic occupations, cleaning workers, beauticians, painters and industrial workers; and some studies pointed to the female gender as a risk factor for this sensitization.^29^, ^30^, ^31^ It is expected a reduction in the sensitivity rates to this preservative in Brazil since the National Health Surveillance Agency (ANVISA, Agência Nacional de Vigilância Sanitária) issued a note stipulating the restriction of MCI/MI concentration at up to 15 ppm in personal products sold in the country, to be complied with before August 2024.^20^, ^37^

Nickel is the main contact allergen in most industrialized countries, with a prevalence of approximately 8% to 19% in adults and a strong predominance in women compared to men (4‒10 times).^32^ Cutaneous exposure occurs from metallic items, detergents, and cosmetics, while systemic exposure occurs through food, water, surgical implants, and dental materials.^32^

Potassium bichromate is present in cement and leather products. It is considered the most common contact dermatitis trigger in civil construction workers and a statistically significant association between sensitization to this substance and male sex has been demonstrated.^33^, ^34^, ^35^

Carba mix is a mixture of rubber-accelerating substances, used in rubber industrial production for shoes, tires and gloves.^36^ Warshaw et al. (2020) have demonstrated that patients sensitized to carba mix were more likely to be male and to have occupational dermatitis compared to individuals who did not react to this allergen.^36^

Concerning hand dermatitis treatment, emollients were prescribed to all patients in the study. These are considered a key component in hand dermatitis treatment, promoting the epidermal barrier recovery and helping to control symptoms.^7^ Topical corticosteroids, which are considered a first-line pharmacological treatment for hand dermatitis, were used in almost all cases.^4^ Topical calcineurin inhibitors can be considered in cases requiring long-term treatment.^5^, ^7^ In the present sample, 31.0% of patients received oral prednisone cycles to optimize clinical control, and oral antihistamines were used in 80.0% of cases, mainly to control pruritus. These findings are similar to what has already been previously described and recommended.^4^, ^5^, ^7^

Of the women studied, 62.0% presented ACD on the hands, while this diagnosis was confirmed in 43.5% of the male patients. The occupational category related to domestic care was the most frequent among patients with ICD and ACD, which coincides with the recent Brazilian literature.^20^ Hand ICD manifested lesions on the palms in 69.0% of the cases. It has been described that irritating eczema is usually limited to exposure sites, generally the palms of the hands and the back of the fingers.^2^, ^5^ However, lesions on the palms, in the present sample, were more prevalent in cases of ACD. The authors emphasize, at this point, the limitation imposed by the data collection from medical records and the difficulty in establishing a relationship between the etiology and clinical findings of HD, which is a disease with a dynamic clinical course.^20^ Lesions on the back of the hands were also more prevalent in cases of ACD, which is in line with the literature since it is pointed out that lesions in ACD are usually well demarcated at the site of exposure – mainly the back of the hands, fingers and wrists.^2^, ^5^

Most of the patients who were discharged from the outpatient clinic after regular clinical control had a diagnosis of ICD. It can be justified by the possibility of controlling the irritant condition with behavioral changes, not depending on the exclusion of one or more specific triggers, as in the case of allergic conditions, which can be more difficult to perform. In fact, there are studies pointing to ACD as a risk factor for a poor long-term prognosis.^3^

In this sample, patients with dyshidrosiform eczema presented a higher percentage of patch test positivity for MCI/MI, nickel sulfate and paraphenylenediamine. The association between dyshidrosiform eczema and sensitization to nickel sulfate and paraphenylenediamine has already been described in the literature.^38^, ^39^, ^40^ This aspect reinforces the importance of doing patch tests in patients with hand eczema.

As limitations, the authors point out the data collection from medical records, which in some cases lacked information, such as DLQI scores, smoking habits and lesions’ precise topography. The frequency of patients with AD may have been underestimated. Furthermore, as this is a cross-sectional study, it was not possible to assess cause-and-effect relationships between the variables.

## Conclusions

This Brazilian investigation of hand dermatitis presents epidemiological, clinical, diagnostic, and therapeutic aspects of the patients. The data reinforces the importance of patch tests in HD investigation and highlights the high sensitivity rates to MCI/MI and nickel sulfate in Brazilian women and to potassium bichromate and carba mix in Brazilian men patients.

Hand eczema is an important condition since its high prevalence and usually chronic course accounts for a significant portion of occupational diseases and compromises patients' QOL. It should be developed health education activities both to prevent the disease and to promote its control as early as possible.

## Financial support

None declared.

## Authors’ contributions

Larissa Relva da Fonte Gonçalves Endlich: Data curation; formal analysis; investigation; methodology; visualization, writing-original draft.

Luciana Paula Samorano: Conceptualization; investigation; methodology; project administration; supervision; validation; visualization; writing-review & editing.

Ricardo Spina Nunes: Formal analysis; investigation.

Vitor Manoel Silva dos Reis: Conceptualization; investigation; methodology; project administration; supervision; validation; visualization; writing-review & editing.

## Conflicts of interest

None declared.
